# Managing a Burning Face: Clinical Manifestations and Therapeutic Approaches for Neurogenic Rosacea

**DOI:** 10.3390/ijms26052366

**Published:** 2025-03-06

**Authors:** Gabriel Aedo, Marco Chahuán, Elsa Gatica, Isabel Herrera, Luis Felipe Parada, Alvaro Seguel, Nigel P. Murray, Sócrates Aedo, Diego Aragón-Caqueo

**Affiliations:** 1 Facultad de Ciencias Medicas, Universidad de Santiago de Chile, Santiago 8320000, Chile; gabrielaedoinostroza@gmail.com (G.A.); chahuanmarco@gmail.com (M.C.); elsa.pscv@gmail.com (E.G.); isabel.herrera@usach.cl (I.H.); luisfelipe.paradaig@gmail.com (L.F.P.); alvaros.86@gmail.com (A.S.); 2 Facultad de Medicina, Universidad Finis Terrae, Santiago 7501015, Chile; nigelpetermurray@gmail.com (N.P.M.); socratesaedo@gmail.com (S.A.); 3 Escuela de Medicina, Universidad de Tarapacá, Arica 1000000, Chile

**Keywords:** neurogenic rosacea, neurogenic inflammation, refractory erythema, rosacea

## Abstract

Rosacea is a common chronic inflammatory condition primarily affecting middle-aged women. It presents with flushing, erythema, telangiectasia, papules, pustules, phymatous changes, and ocular involvement. Although typically grouped into four subtypes—erythematotelangiectatic, papulopustular, ocular, and phymatous—overlapping features often favor a phenotypic diagnostic approach. Neurogenic rosacea (NR) has emerged as a distinct subgroup featuring distinguishing features such as peripheral facial erythema, severe burning and stinging sensations, and resistance to standard rosacea therapies. Recent insights into the pathophysiology of NR propose neural dysregulation as the main driver of the condition. Specifically, the activation of TRP channels at cutaneous sensory nerve endings in the dermis triggers the release of vasoactive peptides, driving neuroinflammation and resulting in burning and stinging. Additionally, there is a marked association with neuropsychiatric comorbidities, which would further mediate the pathogenesis of the condition. In line with this pathophysiological model, NR often fails to respond to conventional rosacea treatments. Instead, patients benefit more from antidepressants and neuroleptic agents that help modulate neuronal activity and alleviate symptoms. This review explores and summarizes the scientific evidence regarding the new insights on disease pathogenesis, clinical manifestations, and proposed treatments for NR.

## 1. Introduction

Rosacea is a chronic inflammatory disease primarily affecting the face and the eyes, characterized by episodes of erythema, papules, pustules, and skin phymatous changes, along with ocular symptoms such as foreign body sensation, photophobia, eye redness, and dryness [1]. It predominantly affects women aged 45–60, with an estimated global prevalence of 5.46% [2]. Its diagnosis relies on clinical presentation, and this condition has traditionally been divided into four subtypes: erythematotelangiectatic (ETR), papulopustular (PP), phymatous, and ocular rosacea [3]. ETR is the most common subtype and typically presents with facial flushing, persistent erythema, and telangiectasia. On the other end, PP rosacea features transient papules and pustules over a central facial erythema, while phymatous rosacea often involves skin thickening and irregular nodular growth, commonly on the nose, chin, forehead, or ears [3]. Ocular rosacea manifests as recurring blepharitis or conjunctivitis, accompanied by periocular erythema, perifollicular eyelid inflammation, and a foreign body sensation [3]. From a clinical point of view, diffuse and persistent facial erythema is the hallmark of most rosacea clinical subtypes [4]. However, whether these subtypes represent a continuum of disease progression or distinct disorders remains controversial [5]. In practice, fitting a patient into one of the categories mentioned above can be problematic, as many features of each clinical subgroup tend to overlap. In this sense, most patients will present with a combination of these clinical subtypes rather than fitting neatly into a single subtype.

To address this complexity, the World Rosacea Consensus (ROSCO 2019) suggested that rosacea should be clinically addressed as phenotypes of disease rather than aiming to classify a patient into a specific clinical subgroup [6]. This consensus established diagnostic phenotypes, major phenotypes, and secondary phenotypes [7,8]. The presence of at least one diagnostic phenotype alone, or at least two major phenotypes, is considered sufficient to make the clinical diagnosis of rosacea [7,8]. Fixed centrofacial erythema in a characteristic pattern with periodic intensification, or phymatous changes of the skin, are regarded as diagnostic phenotypes [9]. Flushing, papules and pustules, telangiectasia, and ocular manifestations are classified as major phenotypes [9], while burning, stinging, edema, or dryness are deemed secondary phenotypes [9,10]. This updated phenotype-based approach provides a more flexible criterion for diagnosing rosacea and offers specific management recommendations based on the predominant phenotype.

Regarding the etiology, rosacea’s pathogenesis is widely acknowledged to be multifactorial, involving genetic susceptibility, innate immune dysregulation, and neurovascular abnormalities [11]. However, the specific molecular basis implicated in the genesis of the disease remains mostly unexplored. It is well documented that several external triggers can precipitate symptoms in susceptible individuals, including the cutaneous microbiome [12], skin barrier dysfunction [13], UV radiation [14], high temperatures [15], stress [16], and hormonal fluctuations [17]. These factors collectively heighten inflammation and neurovascular dysregulation—particularly neuroinflammation, vascular hyperreactivity, and sympathetic nervous system alterations—which contribute to the characteristic erythema and drive the activation of specific inflammatory cascades that determine the formation of papules, pustules, or burning and stinging sensations [18,19,20,21,22].

In this line of work, Scharschmidt et al. [22] proposed neurogenic rosacea (NR) as a novel rosacea subtype, where neuronal dysregulation would play a pivotal role in its pathogenesis and clinical presentation. This dysregulation manifests through vasomotor alterations, neuroinflammation, and neuropathic damage, resulting in intense burning, stinging, and pruritus that often appear disproportionately severe relatively to the degree of the observed erythema [22]. In this subset of patients, a phenotype-based approach might be insufficient, as it may underestimate the burden of the disease and limit therapeutic interventions based solely on the observed phenotype. Moreover, patients with NR frequently have additional neurological and psychiatric comorbidities, such as complex regional pain syndrome, essential tremor, depression, obsessive–compulsive disorder, and post-traumatic stress disorder [6,20,21]. Consequently, the management of NR is inherently difficult and presents significant therapeutic challenges that often require a multidisciplinary approach. In this context, the ROSCO first-line therapy recommendations [6] often show limited results, and alternative therapeutic approaches such as anti-depressants and anti-convulsants have demonstrated greater efficacy in controlling the disease [23].

Despite increasing interest in NR, data on its clinical manifestations and optimal treatment strategies remain limited, mostly relying on case reports and small case series rather than clinical trials or large population-based studies. However, growing clinical and basic science research has unveiled novel neurogenic mechanisms of skin inflammation, further favoring the idea that NR is a distinct entity within the broader rosacea spectrum. In this line, there has been growing consensus that the diagnosis and management of NR require neuropharmacological interventions and multidisciplinary approaches rather than conventional rosacea treatments, as these often fail to achieve adequate disease control and do not address the main underlying pathogenic mechanisms driving the condition. In relation to these novel insights, we conducted a narrative review to consolidate the current knowledge on NR clinical presentation and explore emerging therapeutic options, aiming to enhance its recognition and guide a more effective management for this challenging rosacea variant.

## 2. Methods

A comprehensive literature search was conducted using multiple academic databases and search engines to identify relevant studies for this narrative review. The primary resource was PubMed (including MEDLINE), supplemented by queries in ScienceDirect, Scopus, and Web of Science to ensure coverage of both the biomedical and broader scientific fields. The search strategy incorporated Medical Subject Headings (MeSH) and free-text keywords related to rosacea, neurogenic rosacea, neuropathic pain, neuropharmacology, and skin neurogenic inflammation. Boolean operators (AND, OR) were employed to refine the results, and publications in English from inception through the latest available date were included to capture both historical context and current findings. The articles were initially screened by title and abstract, followed by full-text evaluations to assess methodological quality, completeness of the reported outcomes, and overall relevance. The reference lists of pertinent articles were also examined to identify additional studies not retrieved during the initial database searches.

The review is structured into three main sections to provide a comprehensive overview of NR. The first section explores the pathophysiological mechanisms underlying NR, highlighting emerging evidence on neuroimmune interactions and dysregulated neuromodulatory pathways that contribute to skin inflammation. The second section examines its clinical presentation, detailing key diagnostic features and distinguishing characteristics that set NR apart from other rosacea subtypes. Finally, the third section discusses current and emerging treatment options, emphasizing the role of neuropharmacological interventions and multidisciplinary approaches in achieving better disease control.

## 3. Neurovascular Alterations, Neurogenic Inflammation, and Autonomic Dysregulation in NR

Recent insights into skin innervation and its relationship with the dynamic cutaneous environment have substantially deepened the understanding of how neuroimmune dysregulation can drive and sustain various inflammatory skin disorders, including NR [24]. The skin’s dense neural network, comprised of afferent fibers, unmyelinated C-fibers, myelin-type Aδ fibers, and autonomic nerve fibers, release a broad spectrum of neuropeptides in response to nociceptive stimuli [25,26]. Its primary role is to detect and react to these stimuli, ultimately safeguarding the body against environmental threats [26]. Nevertheless, recent perspectives on how this complex network integrates within the cutaneous microenvironment, as well as how it interconnects with other immune and endocrine pathways, have markedly advanced our understanding of the etiology of various inflammatory skin conditions [27]. Through an intricate interplay, the transduction of neurological impulses from afferent fibers not only transmits nociceptive signals but also modulates local lymphocytes, mast cells, and other immune cells, thereby creating a multidimensional network of interactions [28]. Furthermore, epidermal cells engage closely with neuropeptide-releasing nerve endings and resident immune cells, where cytokines secreted by immune cells can, in turn, provoke additional inflammatory mediators in keratinocytes, perpetuating the inflammatory cascade [29]. In this context, mast cells have gained particular attention. These cells exhibit high upper dermis density, maintaining a direct contact with cutaneous nerve endings and endothelial cells [30]. They can notably become activated by neuropeptides such as substance P, acting as a common bridge between immune system activation and neurogenic inflammation [31]. Upon degranulation, they release an array of pro-inflammatory cytokines and vasoactive amines, amplifying and perpetuating the inflammatory response [32]. In this context, the synergy among the nervous, immune, cutaneous, and endocrine systems relies on a complex communication network of neuropeptides, cytokines, neurotransmitters, small molecules, and other factors such as psychological stress [33]. In this line of work, the skin’s neuroendocrine environment ultimately integrates sensory and hormonal signaling pathways that allow it to function as a peripheral organ system that maintains homeostasis [34]. Beyond serving as a physical barrier, the skin synthesizes and responds to a variety of neurohormones and neuropeptides, among which, corticotropin-releasing hormone (CRH), proopiomelanocortin (POMC)-derived peptides, substance P, and calcitonin gene-related peptide (CGRP) [35]. Keratinocytes, melanocytes, mast cells, and peripheral nerve endings express the requisite receptors for these mediators, creating multidirectional communication loops [36]. This cutaneous neuroendocrine system is highly dynamic. Sensory neurons detect and relay external stimuli, while keratinocytes and immune cells produce trophic or proinflammatory signals in response [37]. Local hormonal feedback mechanisms further refine these interactions, preserving skin homeostasis. In pathophysiological states such as NR, disruptions of these interrelated pathways (ranging from excessive neuropeptide release to autonomic dysregulation), precipitate a pronounced neuroinflammatory response, now recognized as the foundational pillar of disease pathogenesis [37]. Although the concept of neuropeptide–immune–skin crosstalk is longstanding [38], recent work highlights its involvement in the pathogenesis of inflammatory dermatoses, including NR, and supports its emerging role as a therapeutic target.

**Figure 1:**
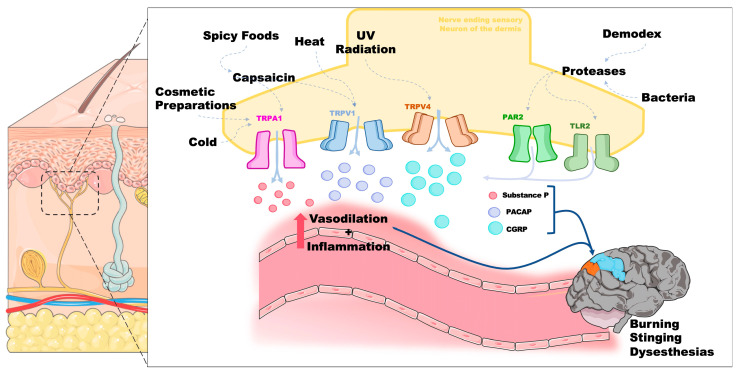
Proposed driver pathophysiological mechanisms in neurogenic rosacea. Activation of TRPA1, TRPV1, and TRPV4 channels on sensory nerve endings triggers the release of vasoactive peptides (substance P, PACAP, and CGRP) into the post-synaptic space, contributing to the symptoms of neurogenic rosacea. The presence of Demodex mites and bacteria exacerbates this condition through the interaction of their proteases with PAR2 and TLR2 channels, enhancing the inflammatory response. Note that the release of vasoactive peptides is not exclusive to any single channel but is depicted in the figure for illustrative purposes. Moreover, these mechanisms have also been previously described in other rosacea subtypes, leading to an overlap of symptoms and clinical presentations.

Patients with NR exhibit heightened sensitivity to a broad range of triggering factors often shared with other rosacea phenotypes, including heat, cold, ultraviolet radiation, and certain cosmetic ingredients. These stimuli activate transient receptor potential (TRP) channels—notably, the ankyrin subfamily (TRPA1) and the vanilloid receptor subfamily (TRPV1 and TRPV4)—on dermal sensory neurons [39,40]. Activation of these TRP channels initiates a cascade of mediators that induce vasodilation in the cutaneous microvasculature, leading to erythema. Specifically, TRP activation leads to the influx of calcium and sodium ions, depolarizing neurons and releasing vasoactive neuropeptides, including substance P and CGRP [41]. CGRP, a potent vasodilator, binds to endothelial receptors, promoting the relaxation of vascular smooth muscle cells and increasing the blood flow to the affected area [42]. Simultaneously, substance P enhances capillary permeability, facilitating plasma extravasation, thus leading to visible erythema [41]. Furthermore, TRP activation stimulates mast cells, prompting their degranulation and subsequent release of histamine and pro-inflammatory cytokines, further amplifying vasodilation and local immune responses [43]. This cycle of neurogenic inflammation perpetuates the erythema, particularly in conditions such as NR, where TRP channel hypersensitivity results in exaggerated and prolonged vasodilatory episodes in response to otherwise mild environmental stimuli. Additionally, it drives the neuroinflammatory process, which signals through ascending sensory tracts to the sensory cortex, resulting in burning and stinging sensations [44,45,46,47]. Unlike other rosacea variants, NR is marked by intense dysesthesia, out of proportion to visible inflammation and erythema, suggesting an underlying neuropathic component. Indeed, NR displays features similar to those of small-fiber neuropathy, wherein dysfunction of small myelinated Aδ fibers and unmyelinated C fibers leads to aberrant nociception, autonomic imbalance, and chronic pain [48]. Excessive TRP channel activation further triggers the release of substance P, CGRP, and pituitary adenylate cyclase-activating peptide (PACAP), which mediate neurogenic vasodilation, increase vascular permeability, and perpetuate a cycle of inflammation [49,50] (Figure 1). Further amplification arises from Toll-like receptor 2 (TLR-2) and protease-activated receptor 2 (PAR-2) signaling on sensory nerve endings and keratinocytes, often elicited by microbial proteases from Demodex mites [50]. The activation of these receptors contributes to maintaining the neuroinflammatory response and creates a positive feedback loop that further increases the release of vasoactive molecules (Figure 1). Autonomic nervous system dysregulation also appears central to NR, as sympathetic overactivity and possible parasympathetic dysfunction foster episodic flushing and heightened vascular reactivity, paralleling mechanisms observed in neuropathic pain disorders such as complex regional pain syndrome (CRPS) [51,52]. Further empirical evidence on the neuroinflammatory and autonomic dysregulation axis of the pathophysiology of NR lies in the observation that standard rosacea therapies often prove inadequate for NR [53]. Instead, given its prominent theorized neuroinflammatory and neuropathic features, agents targeting neuropathic pain pathways—such as gabapentinoids, tricyclic antidepressants, and other neuromodulators—have demonstrated greater efficacy in controlling NR-associated burning, stinging, and flushing [54].

Considering the complex interplay between the nervous, endocrine, and skin systems and the key role of neuroinflammatory pathways in driving symptoms, the next section will explore the clinical presentation of neurogenic rosacea and the comorbidities that often arise alongside it.

## 4. Clinical Manifestations of NR and Its Distinguishing Features with Respect to Other Rosacea Subtypes

NR was first described as a distinct clinical entity through a case series of 14 patients who reported burning sensations, stabbing pain, and facial erythema at levels disproportionate to those seen in patients with other rosacea subtypes [22]. Unlike patients with ETR, in whom the observable erythema is directly linked to vascular changes, NR patients often report significant sensory disturbances even when minimal cutaneous findings are apparent [22]. Although common triggers are shared with the ETR type, including heat, cold, hot water, and psychological stress, the disproportionate reaction to said triggers suggests a heightened neural reactivity and the prominent involvement of nociceptive inflammatory pathways [55]. Additionally, close associations with neuropsychiatric disorders, such as CRPS, depression, obsessive–compulsive disorder (OCD), and post-traumatic stress disorder (PTSD) [16], further favor the role of autonomic nervous system dysregulation in NR pathogenesis. Although NR was originally dismissed as merely a variation of ETR, it has since gained increasing recognition as a distinct clinical entity, drawing special attention due to its unique pathophysiology and symptomatology.

In this sense, the clinical presentation of NR differs markedly from that of ETR (Table 1). Patients presenting with NR tend to take longer to achieve disease remission, with poorer disease control, often exceeding five years, as compared to ETR’s two-year average [5]. Neurogenic symptoms, like intense burning, itching, and ocular discomfort, predominate in NR, while ETR is primarily characterized by persistent erythema and telangiectasia [22]. Another distinguishing feature of NR is its peripheral facial distribution, notably affecting the cheeks and jawline, in contrast to the central face involvement typical of ETR [56]. Interestingly, whereas ETR is exacerbated by heat, patients with NR often find relief in cold exposure [57], further segregating the two subtypes. Another key distinction is the nature of the erythema during disease exacerbations. While ETR and NR share similar triggers, the mechanisms underlying their erythema differ significantly. In ETR, vascular hyperreactivity is the primary driver, whereas in NR, neurogenic inflammation and TRP channel hypersensitivity play a dominant role. As a result, treatments during the acute phase, aimed at reducing vasodilation, such as topical alpha-adrenergic agonists, provide rapid symptom relief in ETR but have little to no effect in NR, for which alternative approaches targeting neurogenic pathways are required [22].

Recent studies suggest that NR shares pathophysiological and clinical features with small-fiber neuropathy, with evidence of C-fiber dysfunction demonstrated by reduced heat pain thresholds in quantitative sensory testing [48]. This neuropathic perspective is further reinforced by the effectiveness of neuropathic pain treatments like gabapentinoids, serotonin–norepinephrine reuptake inhibitors, and tricyclic antidepressants, where traditional rosacea treatments like metronidazole and azelaic acid often fail [58]. On this note, the delayed disease control observed in NR patients compared to those with ETR may be largely attributed to misdiagnosis and, consequently, inappropriate treatment. Because NR is frequently misdiagnosed and treated as ETR, patients often receive therapies that fail to address the prominent underlying neuroinflammatory pathways involved in NR. As a result, treatment fails to yield meaningful improvement, prolonging the time to achieve disease control. In this sense, accurate recognition of NR would steer clinicians toward targeted neuroinflammation-focused interventions, thus reducing the need for trial-and-error approaches with conventional ETR regimens and potentially leading to a more rapid and lasting symptom relief.

On the other hand, the erythema associated with NR can closely resemble a variety of disorders often grouped in a syndromic unit known as the red-face syndrome [59]. This, in turn, warrants a comprehensive differential diagnostic approach, where conditions such as lupus erythematosus, carcinoid syndrome, and pheochromocytoma, among others, must be systematically excluded to ensure diagnostic accuracy [59].

NR distinct spectrum of clinical and symptomatic features, coupled with a pronounced neurovascular pathophysiology, emphasize the importance of recognizing it as a separate clinical entity from ETR. Early identification and interventions that address neuronal hyperexcitability, autonomic dysregulation, and chronic inflammation may substantially impact neuromodulatory pathways and disrupt the cycle of neuroinflammation and response amplification [48]. Conversely, the perpetuation of the inflammatory response may make it even harder to achieve and sustain remission. Moving forward, therapeutic approaches that address the underlying neuropathic and neurovascular mechanisms may provide more durable relief for patients with NR.

## 5. Management Strategies of NR

The available clinical guidelines and consensus recommendations, such as the 2019 ROSCO, primarily focus on treating the visible signs of rosacea while optimizing therapy through an ongoing assessment of symptoms, especially burning and itching [6]. Reducing these symptoms improves the cosmetic outcomes and significantly impacts the overall clinical response and quality of life, which is particularly critical in NR [60]. Recent systematic reviews on phenotypic approaches outline specific therapies for various rosacea manifestations [61], with topical ivermectin showing both notable clinical amelioration and quality-of-life improvement correlate [62]. Systemic treatments, including doxycycline, minocycline, and oral retinoids like isotretinoin, are effective for moderate to severe rosacea by reducing local inflammation and diminishing papules and pustules; however, these interventions generally offer limited control in NR.

As for managing the erythema, systemic beta-blockers have been suggested to reduce the sympathetic effects that drive excessive flushing via beta-adrenergic receptor blockade [63,64,65]. Non-selective beta-blockers, such as carvedilol and propranolol, have been used off-label for the treatment of rosacea-associated erythema and flushing. Although their clinical application is limited by adverse effects, mostly hypotension and bradycardia, their use has shown satisfactory levels of effectiveness in managing persistent erythema and flushing [65]. Carvedilol, in particular, has been titrated in doses of up to 12.5 mg twice daily over three months, showing promising symptom and sign reduction, with a tolerable adverse effect profile [66]. Moreover, carvedilol has been extensively investigated not only for its beta-adrenergic blockade and the consequent reduction in peripheral vasodilation but also for the antioxidant and anti-inflammatory properties of its metabolites [67]. Notably, its metabolites, including carvedilol dihydroxyphenyl and carvedilol O-glucuronide, exhibit potent antioxidant activity and are often regarded as 10 to 100 times more potent than α-tocopherol (vitamin E) [67]. By scavenging reactive oxygen species and inhibiting lipid peroxidation, they counteract oxidative stress [68], a key driver of neuroinflammation in NR. Given NR association with excessive neuropeptide release and TRP channel hypersensitivity, these antioxidative and anti-inflammatory properties may help mitigate chronic neurovascular dysregulation [69]. Additionally, carvedilol’s ability to attenuate sympathetic overactivity and catecholamine-driven vasodilation suggests a dual mechanism that could reduce neuronal sensitization while controlling episodic flushing and erythema in NR [69].

On the other hand, topical alpha-agonists such as brimonidine or oxymetazoline have also been recommended as a viable option, across all levels of erythema severity [70]. However, since erythema in NR emerges from neuroinflammatory pathways rather than reactive vasodilation, these agents often fail to provide the rapid symptom relief seen in ETR patients. Nonetheless, their potential role in modulating sympathetic activity warrants further investigation to clarify their effectiveness in NR management.

Surgical procedures like transthoracic sympathectomy have also yielded improvements in patients with severe flushing, particularly in those with NR, although a small proportion of individuals experience limited efficacy or postoperative complications like reflex sweating [22,71,72]. However, this technique is invasive and costly and has shown variable success rates, which limits its widespread adoption in clinical practice. These factors also restrict further research on its long-term efficacy and safety.

Regarding tailored approaches focusing on neuroinflammation and neurogenic pathways, similar strategies to those employed in CRPS or small-fiber neuropathy have shown promising results [48,73]. Their goals include pain management, neurological modulation, and integrative care. Pain-modulating drugs such as gabapentin and pregabalin can effectively target hyperalgesia and related symptoms, with potential anxiolytic effects as an added benefit [74,75]. These agents bind to voltage-gated calcium channels, reducing neuronal excitability and dampening excessive neurotransmitter release, including of glutamate and substance P [76]. By inhibiting these excitatory pathways, gabapentin and pregabalin address sensory hypersensitivity, which are hallmarks of NR. Additionally, their anxiolytic effects may be beneficial in NR patients, as psychological stress is a known exacerbating factor [75]. Another pharmacological approach involves using neuroleptics and antidepressants. When delivered either topically or systemically, these agents may reduce burning, stinging, and cutaneous hypersensitivity [77]. Neuroleptics exert their effects by modulating dopamine and serotonin receptors, attenuating central dopamine-mediated hyperexcitability and serotonin dysregulation [78], and potentially regulating the neural hypersensitivity associated with NR. In turn, antidepressants act through central pain modulation, enhancing descending inhibitory pain pathways in the central nervous system, thus reducing peripheral sensitization and hyperalgesia [78]. Given that NR is associated with heightened TRP channel activity and autonomic dysregulation, these agents may help restore neurovascular homeostasis by stabilizing neuronal excitability, reducing the exacerbated sympathetic response and potentially interrupting the cycle of neuroinflammation. Coupled with lifestyle modifications, cognitive behavioral therapy, and a multidisciplinary team approach, these interventions can substantially improve patient outcomes [79]. As for specific NR mechanisms, oral medications like gabapentin, pregabalin, and duloxetine may also exert a therapeutic effect by directly inhibiting the TRPV1 and TRPA1 pathways [80,81,82,83]. Duloxetine, in particular, has well-documented success in managing conditions such as complex regional pain syndrome and fibromyalgia, suggesting applicability to NR [84,85]. Although not all studies detail patient-specific dosages, reports describe the complete resolution of NR symptoms in some patients taking pregabalin (300 mg in the morning and 150 mg at night) [55]. Moreover, a multicenter, randomized, double-blinded, placebo-controlled trial on paroxetine in rosacea patients with treatment-refractory erythema showed significant reductions in erythema, flushing, and burning sensations after 12 weeks at a daily dose of 25 mg [86]. Although in this study, the patients were not categorized as NR patients, the inclusion criteria included persistent erythema despite at least three months of tetracycline treatment, with or without the application of topical α-adrenoceptor agonists or intense pulsed light therapy. This refractoriness to treatment, along with the neurovascular components observed in the patients included in the study, make this condition closely resemble NR. Thus, the outcomes reported might be extrapolated to NR for its management, suggesting that paroxetine is a viable and well-tolerated alternative treatment for patients with refractory erythema and also acknowledging the potential neurogenic component of rosacea, given the role of selective serotonin reuptake inhibitors in vascular regulation and their observed benefits in alleviating erythema when other classical options fail to do so.

Beyond these traditional and systemic treatments, recent studies have examined topical TRPV1 inhibitors and botulinum toxin injections. Early research suggests topical TRPV1 inhibitors can effectively inhibit TRPV1 activation, thereby reducing neuronal hyperresponsiveness and relieving the burning or stinging sensations [87]. Moreover, TRPV1 inhibitors have shown favorable results in individuals with sensitive skin, significantly diminishing capsaicin-induced stinging and burning, with adequate levels of tolerability and safety [88]. Additionally, combining topical TRPV1 inhibitors with anti-inflammatory agents such as licochalcone offers enhanced benefits by simultaneously dampening neuronal excitability and modulating inflammatory cascades, such as nuclear factor kappa B (NF-κB) and prostaglandin E2 (PGE2) signaling [89]. This synergy may improve the symptomatic relief while maintaining a favorable safety profile, even with prolonged use. On the other hand, botulinum toxin injections have also shown promise in decreasing facial erythema and flushing [90,91]. The mechanisms by which botulinum toxin may exert its therapeutic effects in NR are yet not fully elucidated. At this level, botulinum toxin is theorized to target key mediators in the neuroinflammation process, blocking acetylcholine receptors and downregulating the secretion of neuropeptides such as substance P and vasoactive intestinal peptide [92]. This, in turn, has a notable effect in regulating vascular hyperreactivity and cutaneous blood flow and also in decreasing the activity of known pruritogenic agents. Additionally, botulinum toxin appears to modulate immune responses by directly inhibiting mast cell degranulation [93]. Mast cells are key mediators in the cathelicidin inflammatory pathway in rosacea, particularly through their role in amplifying the neuroinflammatory response via the upregulation of the antimicrobial peptide LL-37, which plays a central role in the pathophysiology of all rosacea subtypes [94]. Experimental models have shown that botulinum toxin prevents mast cell activation and consequently prevents the amplification of the neuroinflammatory response mediated by mast cells at the local level [93]. From a clinical perspective, intradermal microinjections of botulinum toxin have been shown to decrease erythema, flushing, and telangiectasias for up to three months [91], yet variability in the study designs highlights the need for standardized treatment protocols. The therapeutic approaches discussed, including pharmacologic interventions targeting neurogenic inflammation, autonomic dysregulation, and sensory hypersensitivity in NR, along with dosage regimens, time of onset of response, follow-up duration, study type, clinical outcomes, and potential adverse effects, are summarized in Table 2.

Finally, the therapeutic potential of benzodiazepines and other central nervous system inhibitors has also been explored in the management of NR. Benzodiazepines, functioning as agonists of the gamma-aminobutyric acid (GABA) receptor, exert their effects at the level of both the spinal cord and the peripheral nervous system [95]. By attenuating the sympathetic response and catecholamine release, they may likewise help mitigate the neurogenic features of rosacea [96]. In a similar line, topical applications of agents like ketamine, glycopyrrolate, or capsaicin have shown mixed results [22].

Collectively, these findings illustrate the intricate interplay between neurogenic inflammation, TRP channel dysregulation, and autonomic dysfunction in the pathophysiology of NR. The growing recognition of the neuroimmune crosstalk and its role in sustaining chronic inflammation and vascular dysregulation highlights the need for developing therapeutic strategies centered on targeted neuromodulation. This evolving therapeutic landscape reinforces the increasing consensus that NR should be regarded as a distinct clinical entity, necessitating approaches that move beyond traditional anti-inflammatory and vasoconstrictive measures to effectively address neuronal hypersensitivity and neuropeptide-driven vasodilation.

## 6. Conclusions

NR remains an underrecognized and frequently misdiagnosed clinical entity within the rosacea spectrum. Although it shares overlapping features with other rosacea subtypes, NR is distinct in its clinical manifestations, most notably severe burning and pruritic sensations, along with persistent, refractory erythema, often localized to the peripheral regions of the face. The disease also exhibits a unique association with neuropsychiatric comorbidities, further complicating its diagnosis and management. Traditional dermatological treatments, which primarily target other inflammatory pathways, are often insufficient in NR due to the underlying neurophysiological mechanisms, particularly neuronal dysregulation and neuroinflammation. Consequently, management strategies must incorporate the use of neuroleptics and antidepressants, which target these neural components and address the pain, dysesthesia, and refractory erythema that characterize NR. Training dermatologists to recognize NR as a distinct clinical entity and fostering comfort with integrating neuropharmacological agents into their therapeutic protocols are essential steps toward improving patient outcomes. By broadening the treatment landscape to include these approaches, dermatologists can better manage the complex neurovascular interplay that drives the pathogenesis in NR. Furthermore, a multidisciplinary approach, including collaboration with neurologists and psychiatrists, is crucial in optimizing the use of these treatments and providing comprehensive care. As ongoing research continues to refine our understanding of NR pathophysiology, expanding the role of neuropharmacology in dermatological practice will be central to advancing treatment strategies and enhancing the quality of life of patients with NR.

## Figures and Tables

**Table 1:** Clinical features distinguishing NR from ETR [5,22,55].

	Neurogenic Rosacea (NR)	Erythematotelangiectatic Rosacea (ETR)
**Primary Symptoms**	Burning, stinging, dysesthesias, eye symptoms	Facial erythema, telangiectasia
**Distribution**	Lateral aspects of the face, mostly involving the cheeks	Central face, including the nose and cheeks
**Triggers**	Heat, stress, exercise, menstruation, cold (reduces symptoms)	Heat, sun exposure, spicy foods, alcohol
**Response to Standard Treatments**	Tends to be refractory	Tends to show improvement
**Neurological Symptoms**	Common (burning, stinging, dysesthesias)	Less common
**Neuropsychiatric Disorders**	Frequently co-existing (CRPS, OCD, PTSD, depression)	Less commonly associated
**Treatment (general)**	Beta-blockers, neuromodulators (pregabaline, gabapentin), antidepressants (duloxetine, paroxetine)	Alpha-2 adrenergic agonists, beta blockers, energy-based devices.

**Table 2:** Main therapeutic approaches for NR management.

Intervention	Dosage	Evaluation	Response Time	Time of Follow-Up	Adverse Effects	Type of Evidence	Outcomes
**Gabapentin** [5,48]	Initiated at 300 mg/day, titrated for pain control	VAS, DGA	2–4 weeks	8–12 weeks	Sedation, dizziness, fatigue	CS	Alleviates hyperalgesia and burning; anxiolytic effect.
**Pregabalin** [5,48,55]	300 mg AM + 150 mg PM	VAS, DGA	2–4 weeks	8–12 weeks	Sedation, dizziness, weight gain	CS	Alleviates hyperalgesia and burning; anxiolytic effect.
**Duloxetine** [84,85]	30–60 mg/BID	VAS, DGA, depression rating scales	4–6 weeks	8–12 weeks	GI disturbance, sedation, sexual dysfunction	RCT	Reduces neuropathic pain and erythema.
**Paroxetine** [86]	25 mg/day	DGA	2–4 weeks	12 weeks	GI disturbance, sexual dysfunction, withdrawal	RCT	Improves persistent erythema, flushing, burning, and depressive symptoms
**Beta-Blockers** (Carvedilol, Propranolol) [65,66]	Carvedilol up to 12.5 mg BID; propranolol regimens vary	FSS, DGA	2–6 weeks	8–12 weeks	Bradycardia, hypotension, dizziness	CS, RS	Decrease facial erythema, flushing, and persistent erythema
**Topical TRPV1** **Inhibitors** [87,88]	Trial formulations under study	ESS stinging/ burning rating scales	Immediate–2 weeks	4–8 weeks	Mild irritation, transient erythema	Pilot studies	Rapid reduction in stinging and burning; moderate erythema improvement
**Botulinum Toxin** **Injections** [90,91]	Varies by protocol (units/site)	FSS, DGA	1–2 weeks	3–6 months	Bruising, pain at injection site, possible unintended muscle relaxation	CS, pilot studies	Reduction in facial flushing and erythema; further standardized protocols are needed for broader validation

VAS: visual analog scale; DGA: dermatologist’s global assessment; FSS: flushing severity scale; ESS: erythema severity scale; CS: case series; RCT: randomized controlled trials; RS: retrospective series.

## Data Availability

No new data were created or analyzed in this study. Data sharing is not applicable to this article.

## Abbreviations

The following abbreviations are used in this manuscript:

CGRP	Calcitonin gene-related peptide
CRPS	Complex regional pain syndrome
CS	Case series
CRH	Corticotropin-releasing hormone
DGA	Dermatologist’s global assessment
ESS	Erythema severity scale
ETR	Erythematotelangiectatic rosacea
FSS	Flushing severity scale
GABA	Gamma-aminobutyric acid receptor
NF-κB	Nuclear factor kappa B
NR	Neurogenic rosacea
OCD	Obsessive–compulsive disorder
PACAP	Pituitary adenylate cyclase-activating peptide
PAR-2	Protease-activated receptor 2
PGE2	Prostaglandin E2
POMC	Proopiomelanocortin
PP	Papulopustular rosacea
PTSD	Post-traumatic stress disorder
RCT	Randomized controlled trials
ROSCO	World Rosacea Consensus 2019
RS	Retrospective series
TLR	Toll-like receptors
TRP	Transient receptor potential
TRPA	Transient receptor potential ankyrin subfamily
TRPV	Transient receptor potential vanilloid subfamily
VAS	Visual analog scale
